# Mixed-Methods Systematic Review to Identify Facilitators and Barriers for Parents/Carers to Engage Pre-School Children in Community-Based Opportunities to Be Physically Active

**DOI:** 10.3390/children9111727

**Published:** 2022-11-10

**Authors:** Rachel L. Knight, Catherine A. Sharp, Britt Hallingberg, Kelly A. Mackintosh, Melitta A. McNarry

**Affiliations:** 1 Applied Sports, Technology, Exercise and Medicine (A-STEM) Research Centre, Swansea University, Swansea SA1 8EN, UK; 2 Cardiff School of Sport and Health Sciences, Cardiff Metropolitan University, Cardiff CF5 2YB, UK

**Keywords:** physical activity, sedentary time, sedentary behaviour, youth, socio-ecological mode

## Abstract

**Background:** Low physical activity levels in young children is a major concern. For children aged 0–5 years, engagement with opportunities to be physically active are often driven by the adults responsible for the child’s care. This systematic review explores the barriers and facilitators to parents/caregivers engaging pre-school children in community-based opportunities for physical activity, within real-world settings, or as part of an intervention study. **Methods:** EBSCOhost Medline, CINHAL plus, EBSCOhost SPORTDiscus, Web of Science, ProQuest, and ASSIA were systematically searched for quantitative and qualitative studies published in English between 2015 and 16 May 2022. Data extracted from 16 articles (485 parents/carers; four countries) were quality-assessed using the Mixed Methods Assessment Tool and coded and themed via thematic analysis. **Results:** Nine themes (eight core, one minor) were identified and conceptualised into a socio-ecological model, illustrating factors over four levels: Individual—beliefs and knowledge (and parental parameters); Interpersonal—social benefits, social network, and family dynamic; Community—organisational factors and affordability; and Built and Physical Environment—infrastructure. **Discussion:** The findings provide valuable insights for practitioners and policy makers who commission, design, and deliver community-based physical activity opportunities for pre-school children. Developing strategies and opportunities that seek to address the barriers identified, as well as build on the facilitators highlighted by parents, particularly factors related to infrastructure and affordability, are imperative for physical activity promotion in pre-school children. The perspectives of fathers, socioeconomic and geographical differences, and the importance parents place on physical activity promotion all need to be explored further.

## 1. Introduction

The benefits of being physically active are irrefutable [1]. Unfortunately, physical activity (PA) levels of young children, including pre-school children (0–5-year-olds), are a major concern [2,3]. Specifically, whilst data on this age group are sparse, the emerging evidence is congruent with that of their older counterparts (5–18-year-olds), with high amounts of time in of sedentary behaviour and low levels of PA already established behaviours for many [4,5]. This is especially concerning given that childhood behaviours are well evidenced to track into adolescence and adulthood [6].

For young children, being insufficiently active can limit the protective effect of PA against cardiovascular disease [7] and obesity [8] and have negative connotations for cognitive development [9], psychosocial health [10], and the mastery of fundamental movement skills [11]. Multiple reviews have explored and identified correlates of PA and sedentary behaviour in children (i.e., [12,13]), highlighting that elements relating to parental practices and opinions are, at least to some degree, key determinants of a child’s health behaviours, including PA.

The engagement of pre-school children in organised opportunities to be physically active has been shown to positively influence sedentary behaviour [14] and moderate-to-vigorous physical activity (MVPA) [15]; both important ways to facilitate the achievement of physical activity levels recommended for optimal health [1]. Indeed, Chen et al. [15] found that the participation of 3–5-year-olds in organised sport (outside of school time) was associated with 10% more MVPA throughout the day. Likewise, when evaluating the effects of a 10-week, family-driven active play programme in children under five years old, O’Dwyer et al. [14] discovered that attendance at structured activities away from the home was a significant predictor of lower weekend sedentary time. The authors suggest that this may, at least in part, be due to parental perceptions of PA, and their ability to facilitate the engagement of their child.

The United Nations Convention on the Rights of the Child [16] states that every child has the right to good health and the right to play. However, on an individual level, engagement of pre-school children with opportunities to be physically active is driven by the adults responsible for the child’s care. Tackling physical inactivity and sedentary behaviour in early childhood therefore requires an in-depth understanding of the key factors that influence adults decision-making processes and behaviours. Such information is essential to assist policy makers and, where necessary, address the (mis)perceptions of those individuals who have the greatest influence during the early years period.

A review of global qualitative research synthesised and mapped the factors relating to parental, care provider, and child perceptions of PA behaviour influencers [17]. Seven core themes were outlined, with the most frequently reported barriers and facilitators being those on an interpersonal level, including the role that adults (parents, care providers, family) have in facilitating health behaviours. Given recent evidence surrounding the barriers and facilitators to physical activity engagement among young children [12,17], it is also important to understand how these present for parents/caregivers, specifically in accessing wider opportunities to support their children’s physical activity within local communities. This can provide further recommendations for public health interventions. Consequently, this mixed-methods systematic review aims to build on prior findings by: (i) specifically exploring the barriers and facilitators to parents/caregivers living within developed economies engaging pre-school children (0–5-year-olds) in community-based opportunities for PA, either within real-world settings or as part of an intervention study, capturing both qualitative and quantitative evidence; (ii) categorising and discussing the findings at a socio-ecological level in line with the framework of Sallis et al. [18]; and (iii) establishing which barriers and facilitators may be central to future action planning. From here on, for ease, reference to *parents* will encompass parental, carer, and kinship relationships.

## 2. Methods

This mixed-methods systematic review of both quantitative and qualitative research, including peer-reviewed and non-peer-reviewed (grey) literature, was designed and conducted in line with the Joanna Briggs Institute (JBI) methodology [19]. This approach enabled the complexities of health-related research to be fully explored, ensuring the widest range of understanding was generated [20]. This review is reported in accordance with the Preferred Reporting Items for Systematic reviews and Meta-Analysis (PRISMA) [21] and registered on PROSPERO, registration number CRD42022331738.

### 2.1. Data Sources, Searches, and Study Selection Criteria

Six electronic databases (EBSCOhost Medline, CINHAL plus, EBSCOhost SPORTDiscus, Web of Science, ProQuest, ASSIA), limited to publications in English between 2015 (in line with publication of the United Nations Sustainable Development Goals [22]) and 16th May 2022, were searched by the first author (RLK). Search strategies of key terms were created based on previous reviews of similar topics (e.g., [17]) and refined with guidance from a subject Librarian and input from the review team. The Boolean terms and their variations used included, but were not limited to: (parent* OR carer* OR grandparent* OR guardian*) AND (preschool* OR “early years” OR “young children”) AND (barrier* OR facilitator* OR perception* OR challeng*) AND (“physical activit* OR “active play” OR “fundamental movement” OR “motor skills”).

Two authors (RLK and CS or MAM) independently reviewed all generated citations and abstracts to select eligible studies using Rayyan (QRCI, Qatar), with those coded ‘include’ subsequently full-text screened (independently by RK and MM) against the pre-defined inclusion/exclusion criteria (Table 1). Disagreements regarding eligibility of citations and abstracts or full-text articles were resolved through discussion between the authors (initially: *k* = 0.46, after discussion: *k* = 0.88), with a third reviewer (CS or BH) consulted where a consensus could not be reached (*n* = 3). Online File S1 provides full details of database-specific terms, restrictions and search strategies, and secondary and grey literature search processes.

### 2.2. Data Extraction

A standardised form pre-developed by the JBI (mixed methods convergent integrated approach) [19] was used by RLK to extract data from the included studies for evidence synthesis. Extracted information included: authors; year of publication; study design, setting, and population, including sample size; participant demographics and characteristics; study methodology; inclusion/exclusion criteria; empirical and/or contextual results relating to parental/carer perceived barriers and facilitators to engagement with community-based PA opportunities (for quantitative data: outcomes of descriptive and interferential analyses; for qualitative: themes or sub-themes supported with illustrations, such as supporting data or direct quotations); type of community-based opportunity (if applicable); and quality-assessment information. A second reviewer (CS or BH) independently reviewed 50% of the extracted data. Two discrepancies were resolved through discussion. Supplementary data were consulted where necessary and available.

### 2.3. Quality Assessment

One author (RLK) independently assessed study quality using the Mixed Methods Assessment Tool (MMAT) [23]. A second reviewer (CS or BH) independently randomly checked 50% of the studies to ensure consistency. No disagreements occurred. Each study was subsequently attributed an overall quality score, ranging from 1*, where 20% of the quality criteria have been met, to 5*, where 100% of the quality criteria have been met [24]. No studies were excluded due to low quality; rather, all issues were considered when interpreting the results of each study. As per JBI recommendations for mixed-methods systematic reviews, an assessment of certainty was not conducted.

### 2.4. Data Synthesis

A convergent integrated approach to data synthesis was undertaken [19]. Quantitative data were transformed into textual descriptions or narrative interpretations to facilitate integration with extracted qualitative data. The transformed quantitative data were concurrently analysed and categorised with the qualitative data to form one set of themes and narratives. Thematic synthesis of the pooled data was conducted by RLK following the approach of Thomas and Harden [25]. The findings were coded line-by-line with ‘free codes’, organised into related areas, and ‘descriptive’ themes constructed. The descriptive themes were organised into overarching ‘analytical’ themes. This process was completed in collaboration with the review team; generated themes were discussed, reflected upon, and refined, with reference back to the original articles when required. The analytical themes were used to construct a conceptual framework based on the socioecological model categories outlined by Sallis et al. [18]. This process was undertaken by RLK with a ‘critical friend’ (CS) blindly crossmatching 10% of the studies against the framework to ensure the accuracy, rigour, transparency, and credibility of the undertaken process [26].

## 3. Results

**Figure 1:**
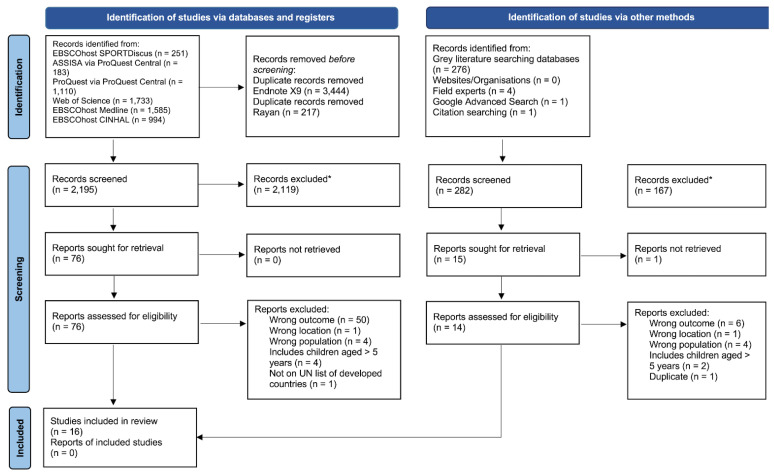
PRISMA 2020 flow diagram for new systematic reviews which included searches of databases, registers, and other sources. Note: * Automation tools were not used; *n* = number; UN = United Nation.

Overall, 5856 articles were identified from electronic database searches; a further 282 were identified from secondary search strategies. Following duplicate removal, 2477 articles were screened, 2286 excluded, 91 retrieved for full-text eligibility screening, and 16 retained and included in the final analysis (Figure 1). The remaining articles included data from 485 parents of a child aged 0–5 years old [27,28,29,30,31,32,33,34,35,36,37,38,39,40,41,42], from four different countries (Australia, the United States of America (USA), the United Kingdom (UK), and Slovenia). Three articles did not provide details of the age of parents [28,32,40]; one article did not provide the sex of parents [38]. It was not possible to separate core demographic details from other study participants not relevant to this review within four articles [31,38,41,42]; however, given that it was possible to separate the facilitators and barriers, these studies were still included.

All articles collected qualitative data, predominantly via semi-structured interviews (*n* = 9/16) [27,28,29,30,31,32,33,34,36], with five articles adopting a mixed-methods design [28,29,34,40,42]. From these five articles, only the quantitative data from one article [28] met the criteria for inclusion in the data synthesis and was assessed for quality. Only 5 out of 16 articles (31%) had the primary aim to explore factors associated with the child’s engagement in community-based opportunities for PA [27,28,31,34,42]. For the others, the aim was either focused on the general PA context (*n* = 8/16) [32,33,35,36,37,38,40,41] or on other health-related behaviours (*n* = 3/16) [29,30,39], with data pertinent to this review captured as a coincidental finding. Further individual study characteristics and overall MMAT quality scores are provided in Table 2, with a full breakdown of the quality assessments and supporting justification presented in Online Tables S1 and S2.

**Figure 2:**
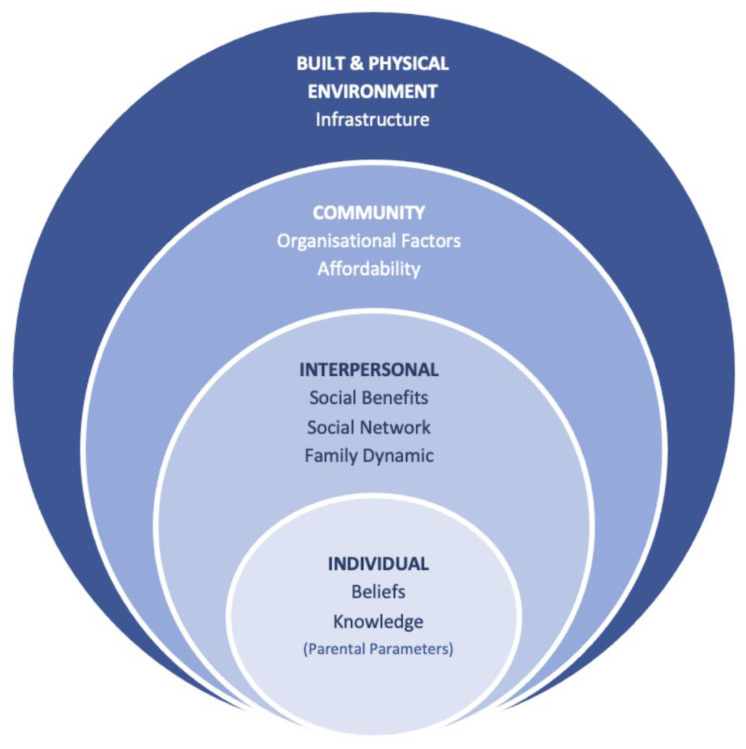
Socio-ecological model of factors that influence parental engagement of pre-school children in community-based opportunities for physical activity.

In concert with the dimensions of the socio-ecological framework of Sallis et al. [18], a narrative synthesis of the findings is outlined in the following section. To assist with framing potential interactions between individual-level parental influences and wider factors [18], this is accompanied by a model conceptualised from the nine themes (eight core, one minor) identified within the analysis process (Figure 2). An overview of the themes with indicative text quotes is presented in Online File S2.

### 3.1. Individual

#### Beliefs and Knowledge (and Parental Parameters)

At an intrinsic level, parental *beliefs* regarding the value they place on PA, and the wider benefits they perceive it could bring, appear central to their decision-making processes. Structured PA was perceived to provide exposure to stimulating learning opportunities and environments [27,42] and to foster a broad range of positive and healthy child-development traits. In addition to being an approach to develop social and emotional skills [27,28,31,42], companionship [28], confidence [42], and teamwork [27] were also referred to. Specifically relating to enrolment in private swimming lessons, participation was viewed as a way for children to gain an essential life skill (personal safety), and an early childhood education that would be advantageous in the longer-term [27]. For parents classified by studies as middle-class, swimming lessons were also perceived as a way to demonstrate ‘good parenting’ by enabling them to comply with social norms and mitigate their own concerns regarding fear of drowning and water safety [27].

Perceived benefits were also deemed to be derived from professional instruction. Specifically, knowing a professional would be present in one study facilitated fathers’ engagement with an active play intervention [34], whilst the anticipated development of strong, sport-specific skills due to instruction motivated attendance at bouldering classes [31]. Paying for skilled instruction and access to an appropriate role model that would enhance a child’s behaviour and subsequent learning, facilitated enrolment in private swimming lessons [27]. Indeed, an individual, versus a group-based approach, was considered to be associated with even greater gains [27].

Closely linked to beliefs, parental *knowledge*, either prior knowledge or a lack of, influenced their decision making. At a basic level, not having the skills to teach their child to swim themselves facilitated attendance at private swimming lessons [27]. However, the recognition and understanding that early skill development can be beneficial for securing leisure opportunities in later life [27] and bring health and fitness benefits in the short term [31] may still be offset by other factors. For example, Houghton et al. [34] found a misconception among fathers that available sessions, including toddler groups, were purely for mothers, reflecting how lack of knowledge can be a barrier to attendance. Briefly, although not a major finding, it is pertinent to note that factors relating to *parental parameters,* namely, health and personality traits (shyness, laziness), were also highlighted as potential barriers to engagement by fathers attending community-based active play sessions as part of an intervention study [34].

### 3.2. Interpersonal

#### Social Benefits, Social Networks, and Family Dynamics

A key driver for attending playgroups [28] and parks [33] was the opportunity they provide for parents [28,42], particularly mothers [33,37], to *socialise*. Moreover, *social networks* provide a platform for peer support and influence. Playgroups provide a sense of togetherness, where comradeship can thrive, parenting practices can be shared in a safe space, and observations made of other parent–child interactions to inform one’s own future parenting practices [30]. Social networks facilitated the use of parks [33] and other PA opportunities through the sharing of knowledge and resources, for example, attending in groups or sharing transport [37]. Where such peer influence had a predominantly positive impact, including participation on the basis that ‘everyone else is doing it’ [27], differing parenting approaches and/or attitudes, particularly towards choice of leisure time activity (café versus park), may act as a barrier to pre-schoolers’ PA [33].

Challenges can also arise from within the family unit that influence the *family dynamic* and, subsequently, PA engagement. Parents in general, and fathers specifically, highlighted the difficulties that arose from having children of different ages. Specifically, competing child commitments [33] and having children outside the age-range of the targeted class [34] were raised as barriers to engagement. Moreover, while mothers often delegated the teaching of new sports to fathers or professionals [40], they were, however, often the ‘gatekeeper’ to engagement in PA opportunities, self-perceiving that they were the drivers of initiating new activities [40] and perceived by fathers to be the ‘organiser’ and reason for their attendance and co-participation at activities [34].

### 3.3. Community

#### Organisational Factors and Affordability

When targeting PA in pre-schoolers, the unique requirements of this population and their parents should be acknowledged at an *organisational* level. Age-group-targeted opportunities that are developmentally appropriate, based on the principles of learning through play, and provide a different experience (e.g., bouldering classes) inspired parents and facilitated attendance [31]. Moreover, a lack of professionally supervised activities for this age group acted as a barrier [37]. The timing of community-based opportunities was also important. Holding more events on weekends [33] could increase engagement; commitment and schedule clashes were barriers identified by both mothers [37] and fathers [34]. For opportunities specifically targeting the engagement of fathers, Saturday sessions led to the greatest attendance levels [34].

The engagement of parents could, in part, be facilitated or restricted by advertising strategies. Social media was identified as a tool for sharing PA ideas and local events [33]. However, it was noted that whilst improved advertising of community PA opportunities could facilitate greater co-participation of parents with their child(ren) [33], poor advertising was a barrier that led to a lack of understanding about available sessions [34]. Being used in an alternative format, marketing techniques delivered through social media that highlighted the perceived threat of not being able to swim was an effective way of encouraging parents to enrol their pre-schooler in private swimming lessons [27].

A major barrier for parents was the affordability of PA. The high cost of participation in organised sports or structured activities (e.g., ice skating) limited engagement [36,37,38,39,41]. For families in deprived areas, the cost of, for example, general swimming, let alone private swimming lessons, precluded participation [41]. Despite wanting to enrol their child(ren), for Brazilian immigrant mothers and fathers in the USA, it was simply not an activity they could readily afford [36,37]. For those with limited finances, a lack of disposable income was a significant barrier to pre-schoolers’ PA [36,41], whereas for middle-class parents, being able to afford the additional cost of private swimming lessons was not something they were concerned about [27].

The provision of low-cost and free activities was viewed as an approach to incentivise participation, particularly for those living in deprived areas [41]. Improving access to resources already available in the community (school facilities) and greater support from community councils were highlighted as ways to improve PA opportunities for young families [33]. Additionally, where fathers identified the limited availability of affordable organised sport as a barrier to their pre-schoolers’ PA [36], individuals embracing or local providers facilitating opportunities to engage in locality-specific activities (bushwalking) [33] could provide a viable solution.

### 3.4. Built and Physical Environment

#### Infrastructure

The *infrastructure* surrounding adequate access to parks, open spaces, general resources, their quality, and how easy it is to travel to them presented an important facilitator of play, and therefore PA, for pre-schoolers. A lack of available, well-resourced facilities and/or parks with age-appropriate equipment [28,33], all-weather provisions [29,33], and adequate green space [36] were mentioned as hinderances. Access to open space was somewhat more of an issue in outer, than inner, Australian suburbs [28], whilst, in some rural farmland areas of the USA, parks were reportedly non-existent [32]. In addition to a lack of access to outdoor facilities, the availability of indoor facilities, particularly in more rural areas [29], was also highlighted. Parents would like greater access to indoor play centres [29] and programmes and services, such as local events and playgroups [33].

Even when access is not an issue, the quality and safety of available facilities was often raised, particularly in parks [28,35,39]. Deteriorating conditions, where broken and removed equipment has not been replaced [28], safety hazards (broken glass and equipment) [39], the presence of dogs within park areas, and safety issues associated with traffic and park locations [28], all deterred parents from taking their preschool-aged children. Similarly, these issues were highlighted to a greater extent in outer rather than inner suburb areas [28].

Inherently linked are issues surrounding distance and transportation. Whether in rural or urban areas, the distance parents have to travel to attend parks, green spaces, and other facilities and opportunities that encourage play and PA had a significant impact on their frequency of attendance [29,32,33,36]. In more rural farmland areas [32], outer suburbs [28], and areas of high deprivation [41], attendance is often governed by access to transport, whether public or private. With this comes the associated cost of fuel [38] or fares, and sometimes excessive and unmanageable travel times [32].

### 3.5. Discussion

This mixed-methods systematic review examined the facilitators and barriers to parents engaging pre-school children in community-based PA opportunities and environments. To support the interpretation and translation of the findings, the results of the review were categorised using a socio-ecological approach (Figure 2). From the 16 included articles, eight core themes were identified over four levels: (i) *Individual*—belief, knowledge; (ii) *Interpersonal*—social benefits, family dynamic, and social network; (iii) *Community*—organisational factors and affordability; and (iv) *Built and Physical Environment*—infrastructure.

Parental beliefs regarding the value of PA and their knowledge around its benefits influence engagement with community opportunities. Beliefs, which focused on the positive development of traits and experiences that could be acquired by the child, act as a facilitator [27,28,31,42]. However, limited knowledge, whether through lack of skills to teach their child themselves or about specific PA opportunities, can simultaneously facilitate [27] and prohibit attendance [34]. At an individual level, although not a core theme, perhaps due to limited studies, the influence of parental parameters was also highlighted. Given the limited evidence to support this theme, more detailed discussions are precluded.

The importance of personal growth resonates within the existing literature on parental motivations for supporting pre-school children’s leisure activities. For example, higher levels of behavioural engagement in leisure activities, which is frequently organised sport, presents more often among parents who value leisure activities for their ability to shape children’s competencies and provide enjoyment [43]. There is also a strong relationship between greater participation in leisure opportunities for children and more favourable family economic factors [44]. Given, however, the strong relationship between the accessibility of opportunities in communities to be physically active and family resources, such as time, money, and location, as evidenced in this review, these are likely to present as more prominent barriers for less affluent families.

It is important to highlight that the values placed on leisure-time use may vary among parents of different social classes [45]. Previous research has identified less affluent parents as being less likely to believe that structured leisure activities may help children overcome social and behavioural difficulties [43] and, in contrast to more affluent families, to place more emphasis on safety and opportunities for free time (as opposed to structured activity) [46]. Considering parental motivations and values is central to shaping future opportunities for parents of young children, as well as reducing health inequalities. How these motivations and values are associated with family affluence within local communities warrants further research.

Social norms are evolving; whilst, historically, mothers have been the primary-care providers for children, changes in policy over recent years have advocated that it should no longer be the default. For example, in 2015 in the UK, Shared Parental Leave was introduced [47]. This change in the provision of primary childcare impacts the landscape of the household and family dynamic roles; with fathers playing a more central role at home, it is important to consider at whom opportunities that facilitate PA are targeted (mainly mothers) [34]. Indeed, the social networks and benefits that parents [28,42], and particularly mothers [33,37], obtain from taking their children to activities drive both their initial engagement with community-based PA opportunities for their child and their sustained attendance. Whether such factors equally motivate fathers is unclear from the current evidence base.

The Capability, Opportunity, Motivation, and Behaviour model (COM-B) [48] proposes that three factors (*Capability*, *Opportunity,* or *Motivation*), either combined or in isolation, can facilitate health-related behaviour. In this instance, considering *Motivation,* whilst parents might initially be driven to attend community-based opportunities for PA for their child’s or for their own benefit (e.g., personal social benefits), observing their child’s enjoyment through being active and having fun may subsequently lead to bi-directional effects, with positive changes in parental beliefs leading to more engaged participation from the child [43].

When considering *Opportunity*, the direct cost of activities or cost of getting to locations (e.g., parks) [36,37,38,39,41] and infrastructure-based limitations [28,29,32,33,35,36,39] both presented as major barriers to participation. In the UK, at a time when planning departments are now more heavily involved in creating infrastructures that encourage PA, these issues should be ones that can be alleviated. Additionally, current policies and developments in active education settings [49] support the use of pre-existing local community facilities, such as schools [33], as venues for PA opportunities, minimising both cost and travel requirements, but also building community cohesion.

### 3.6. Future Directions

Multiple factors across multiple different socio-ecological levels that parents identify as influencing the engagement of their pre-school child in community-based opportunities for PA were highlighted. Whilst these provide key areas for practitioner and policy-maker consideration, for some, more research is required to further understand their impact. First, the results indicate gender differences in parental perceptions of barriers and facilitators. Whilst the views and needs of fathers are discussed within some of the included articles, the percentage of female study participants is substantially greater (Table 2), and drivers of engagement potentially differ. For example, playgroups and parks provide opportunities for mothers to socialise [33,37]; however, this needs to be explored further from a male perspective. Dual-advertising campaigns may be needed to target the parenting roles simultaneously and widen participation and engagement to the whole family unit.

Second, it is apparent that the factors that may have the greatest influence on parents who are more or less affluent and/or live in rural, versus urban, locations might differ. Where affordability and access barriers present the primary challenge in areas with higher deprivation (i.e., [29,32,36,37,39]), for parents where cost is not perceived to be an issue, influencing factors appear to be situated at an individual or interpersonal level (i.e., [27,28,31,33,42]). Given the limited number of studies that have specifically set out to explore this in parents of pre-school children, studies that compare such differences in greater depth within local communities are required. Proportional universalism [50], where policy implementation strategies and provision are provided to all, but provision is graded dependent on need, is required.

Finally, concerning parental beliefs and knowledge, further work is needed to ascertain where on the spectrum of importance parents place engaging pre-schoolers in PA. Conventionally, it has been perceived that pre-schoolers are sufficiently active by default (as supported by the findings of Hesketh et al. [17]). However, with emerging evidence to show that this is not the case, with high levels of sedentary behaviour and low levels of PA potentially already established in some (i.e., [3,5]), this pre-conception needs to be urgently challenged.

### 3.7. Strengths and Limitations

This review was designed to take a systematic approach, using rigorous methods and validated tools, following a pre-defined protocol. Positively, the participant samples, in combination, represent a broad range of the target population encompassing males, females, ethnical diversity and different levels of socio-economic status. However, it is still possible that some studies containing pertinent data may not have been captured. It is important to note that only studies published in English from one of the 37 countries listed on the United Nations 2022 list of developed economies [51] were included, thus limiting the extrapolation of findings to other countries. Future research should explore and consider the specific environmental and cultural parameters and constraints of less developed economies. Additionally, the quality of included studies varied. Whilst 11 met 100% of the MMAT quality criteria [30,31,32,33,34,36,37,38,39,41], two articles only met 40% of the criteria [28,40], one met 60%, [42] and two met 80% [27,29]. It also became apparent during the latter stages of the screening processes that studies containing the views of expectant parents (terminology not agreed and included within the search terms by the team and its wider collaborators) may have provided additional insight. As per the pre-defined criteria, within this review, such studies (e.g., [52]) were duly excluded from the final analysis. It is important to additionally highlight that the results for the theme ‘Beliefs and Knowledge’ are largely based on the findings of a single study [27], potentially introducing an element of bias. Therefore, this needs to be considered when interpreting the results.

## 4. Conclusions

Physical inactivity is detrimental to health and well-being across the lifespan, making the embodiment of healthy behaviours from the early years phase essential. The message from PA guidelines is consistent and has not changed since 2011 [53]; therefore, identifying ways to help parents realise the potential of early PA is important. The findings within this evidence synthesis provide valuable insights for practitioners and policy makers involved in commissioning, designing, and delivering community-based PA opportunities for pre-school children. To tackle physical inactivity and promote PA, developing strategies and opportunities that acknowledge and seek to address the barriers identified and build on the facilitators highlighted by parents, particularly surrounding infrastructure and affordability, may be paramount.

## Figures and Tables

**Table 1:** Study inclusion and exclusion criteria.

Variable	Inclusion Criteria	Exclusion Criteria
Population or participants	Parent/guardian/carer of a child(ren) aged 0–5 years Any gender Restricted to the 37 countries listed on the UN 2022 list of developed economies	Studies including the parent, guardian, or carer of children aged >5 years, where data cannot be separated Studies that include clinical populations (e.g., autism, cerebral palsy, cystic fibrosis), where data cannot be separated
Phenomena of interest	Perceived parental, guardian, or carer barriers/facilitators to engagement of children aged 0–5 years with PA driven opportunities	Studies including the perceptions of children or education providers Studies that involve data obtained relating specifically to the effects of COVID-19 pandemic curtailment restrictions
Context	Community-based PA opportunities—any public, private, or third-sector activity provided either freely or following payment within real-world settings or as part of an intervention study	Studies where a degree of activity offered (free or paid) is not based on or driven by PA Studies that report outcomes relating to physical education provision or interventions delivered during core nursery and school hours and therefore participation is not under the direct influence of a parent/guardian/carer
Study designs	Any quantitative, qualitative, or mixed-methods study design providing original results (including grey literature, such as conference proceedings) No restrictions on measurement type for assessing barriers/facilitators. Mixed-methods studies will be considered if the data from the qualitative and quantitative elements can be clearly extracted	Studies not providing original results, such as systematic reviews, meta-analysis, general reviews, or editorials Studies where barriers/facilitators are only reported anecdotally within the discussion, not as core results

PA = physical activity; UN = United Nations.

**Table 2:** Study characteristics and MMAT scores.

Author	Study Design	Location and Context	Inclusion/ Exclusion Criteria	Phenomena of Interest	Participants (*n*)	Core Characteristics		Overall MMAT Grade
						Parent	Child	
Allen et al. (2021) [27]	Descriptive via semi-structured interviews (random sample)	UK: North-east of England Private /franchised swimming lessons	Aged ≥ 18 years; previously paid for private swimming lessons for child(ren) (0–4 years) for 12 months	The reasons why middle-class parents decide to pay for private/franchised swimming lessons for pre-school children in line with Bourdieu’s triad of capital, habitus, and field	*n* = 8 (75% female) Aged 25–44 years	Education level 50% degree 25% college 25% secondary school Employment 50% employed 12.5% unemployed 12.5% homemaker 25% other Income 25% low-middle-class 75% mid-middle class	Aged 0–4 years	****
Andrews et al. (2019) [28]	Mixed methods Stage 1: Cross- sectional via individually constructed self- report questionnaire (stratified purposive sample) Stage 2: Descriptive via semi-structure interviews (self-selected from Stage 1)	Australia: Melbourne Selected inner and outer-suburban areas, one > 25 km and one < 10 km from the central business district	Lived in their suburb for ≥12 months; parent of at least one child aged 0–4 years attending pre-school	Who children played with and where they played in the two communities and the reasons for any differences in children’s play experiences	Inner suburbs Questionnaire *n* = 72 (95.8% female) Interviews *n* = 10 Outer suburbs Questionnaire *n* = 26 (92.3% female) Interviews *n* = 10 No age range detailed	Inner suburbs 77.8% born in Australia Education level 34.4% postgraduate 37.5% degree 2.8% year 12 not completed Employment Hours/week paid work Mean = 16 (σ 14.6) Outer suburbs 46.2% born in Australia Education level 15.4% postgraduate 34.6% degree 11.5% year 12 not completed Employment Hours/week paid work Mean = 10.2 (σ 15.4)	Aged 0–4 years	**
Downing et al. (2016) [29]	Mixed methods. Descriptive via structured interviews (only qualitative data relevant; purposive sample)	Australia: Victoria Urban and regional local government areas covering the lowest decile for socioeconomic disadvantage	Mother of child(ren) aged 1–3 years; from low SES urban or regional area; able to speak, read, and write fluent English	The views of mothers from disadvantaged (low SES position) urban and regional areas (e.g., beyond major capital cities) as potential end users of child active play and screen time behaviour change interventions, with a focus on text messaging and web-based delivery platforms	Urban *n* = 22 (100% female) Mean age = 33.9 years (σ 6.4) Regional *n* = 10 (100% female) Mean age = 32.4 years (σ 5.7)	Urban Education level 46% degree or higher 46% year 12/trade/diploma 9% year 10 or equivalent Employment 60% employed 23% home duties 18% student/unemployed/other Regional Educational level 50% degree or higher 30% year 12/trade/diploma 20% Year 10 or equivalent Employment 30% employed 50% home duties 20% student/ unemployed/other	Mean age 2.5 years (σ 0.9) Lived with mother all/most of time	****
Fuller et al. (2019) [30]	Descriptive via semi-structured interviews (purposive sample)	Australia: Greater Brisbane Metropolitan area. Playgroup attendees	Parent of child attending a playgroup that agreed to hold a focus group	Barriers/facilitators to using parenting practices that support healthy obesity- related behaviour development in their child and what is acceptable in terms of intervention delivery mode and timing. Findings to inform the design of a community playgroup childhood obesity prevention intervention	*n* = 30 (93.3% female) 1 father 1 grandmother 13% aged > 30 years 37% aged 30–35 years 50% aged 36+ years	Education level 50% university 40% TAFE or trade 10% secondary school Employment 50% employed 50% unemployed	Median age = 24 months	*****
Gridley (2022) [31]	Descriptive via semi-structured interviews (purposive sample)	UK: England Indoor climbing centres	Parents with a child who had attended 2+ sessions of a developmentally adapted indoor bouldering programme	The perceptions of parents whose children took part in a developmentally adapted indoor bouldering programme designed for < 6-year-old children	*n* = 6 overall Mean age = 35.5 years (σ 6.4) Derived from text that *n* = 3 attended rock tots (33% female)	Unable to separate any details from those of parents who attended rock kids (children aged > 5 years)	Aged 1–4 years (rock tots)	*****
Grzywacz et al. (2016) [32]	Descriptive via semi-structured interviews (part of a larger study; purposive sample)	USA: North Carolina Latino farmworker families	Female with child aged 2–5 years; household member employed in agriculture in the last 12 months	The beliefs held by mothers in Latino farmworker families about the contribution of PA to pre-school-aged children’s health and the perceived barriers or constraints that impose limits on pre-school-aged farmworkers’ children’s PA	*n* = 33 (100% female) No age range detailed	n = 16 migrant families n = 17 seasonal families Education No details Employment 40% worked on farms during past 12 months	Aged 2–5 years	*****
Hnatiuk et al. (2020) [33]	Descriptive via semi-structured interviews (purposive sample)	Australia: Western Sydney	Aged ≥ 18 years; parent of child aged 2–4 years	Parents’ perceptions about: (i) PA and possible benefits of family-based co- participation in PA, (ii) the perceived facilitators and barriers to co-participation in Western Sydney, and (iii) recommendations for improving co- participation within their community	*n* = 15 (93% female) Mean age = 34.9 years (σ 5.4)	73% born in Australia Education level 20% postgraduate 40% undergraduate degree 27% trade certificate or diploma 13% year 12 or equivalent Employment 53% employed 6% student	Aged 2–4 years Mean age = 3.5 years (σ 0.56)	*****
Houghton et al. (2015) [34]	Mixed methods. Descriptive via structured interviews (only qualitative data relevant; purposive sample)	UK: Liverpool Sure Start Children’s Centres	Fathers/male carers of child aged 3–5 years; living within catchment area of 26 Sure Start Children’s Centres; attended at least 4 out of 6 sessions	The effectiveness and feasibility of a physically active-play-based programme on fathers’ engagement with their pre-school-aged children across Liverpool	*n* = 36 95.7% fathers; 3.2% grandads; and 1.1% other Mean age = 37.7 years (σ 8.7)	70.2% White British 26.6% had not used Sure Start service before Education level No details Employment 56.4% employed 33% unemployed 4.3% retired 3.2% full time students 3.2% unable to work	Mean age = 3.8 years (σ 1.2)	*****
Joseph et al. (2019) [35]	Descriptive via focus groups (part of a larger longitudinal mixed-methods study; purposive sample)	USA: Louisiana Early care education centres	Legal guardian/caretaker of child aged 3–5 years enrolled in ECC; understand and speak English; free from cognitive impairment; parent not an ECC provider; child not at ECC assessed for longitudinal study arm	The views and differences between parent and ECC provider perspectives on barriers/facilitators to children’s PA and screen time, awareness of relevant regulations and recommendations, and caregiver- identified opportunities to leverage screen time to increase young children’s PA	*n* = 28 (94% female) Age range 24–59 years	67% African American (Data for n = 18) Education level (Data for n = 18) 22% > college degree 22% some college/college degree 56% ≤ high school diploma Employment (Data for n = 16) 69% employed 31% unemployed/retired	Mean age = 3.7 years (σ 0.8)	*****
Lindsay et al. (2022) [36]	Descriptive via semi-structured interviews (part of a larger longitudinal mixed-methods study; purposive sample)	USA: Massachusetts Brazilian immigrant families	Father of at least one child aged 2–5 years; Brazilian ethnicity and born in Brazil; aged ≥ 21 years; lived in Massachusetts; lived in the USA ≥ 12 months	The perspectives and parenting practices of Brazilian immigrant fathers with regard to their pre-school-age children’s PA and sedentary time	*n* = 21 (100% male) Mean age = 34.4 years (σ 2.8) Age range 27–43 years	Education level 4.7% college 71.5% high school/high school diploma 19.1% < high school Employment 100% employed	Aged 2–5 years	*****
Lindsay et al. (2019) [37]	Descriptive via focus groups (part of a larger longitudinal mixed-methods study) (convenience sample)	USA: Greater Boston area, Massachusetts Brazilian immigrant families	Mother of at least one child aged 2–5 years; Brazilian ethnicity and born in Brazil; lived in the USA for ≥12 months	PA parenting practices used by Brazilian immigrant mothers of pre-school-aged children	*n* = 37 (100% female) Mean age = 35.3 (σ 2.8) Age range 26–41 years	Education level 56.8% graduated high school Employment 92% owned their own housecleaning business	Aged 2–5 years	*****
Martin-Biggers et al. (2015) [38]	Descriptive via focus groups (part of a larger longitudinal mixed-methods study (purposive sample) Each focus group randomly asked about 2 different topics from a selection of 7)	USA: New Jersey and Arizona	Parent of child aged 2–5 years; primary language English or Spanish	Parents’ cognitions associated with key obesity-prevention behaviours	*n* = 28 (active -play/barriers focus groups) No sex demographics supplied	Unable to extract focus-group-specific data	Aged 2–5 years	*****
Penilla et al. (2017) [39]	Descriptive via focus groups (purposive sample)	USA: San Francisco Community-based organization in the Mission District	Parent/guardian of a child between 2 and 5 years; English- or Spanish-speaking; of Mexican, Guatemalan, or Salvadoran descent	The broader social and environmental influences, namely, how urban neighbourhoods influence children’s weight status through parents’ child-feeding and PA routines	*n* = 49 (55% female) Mean age mothers 30 years (σ 5.8) Mean age fathers 35 years (σ 9.1)	Mothers Education level Mean years in education 12 (σ 3.7) Employment 33% currently employed Fathers Education level Mean years in education 11 (σ 2.3) Employment 68% currently employed	Aged 2–5 years	*****
Pišot (2020) [40]	Mixed methods Descriptive via structured interviews (only qualitative data relevant)	No details: Presume Slovenia	Mother of child aged 4 years Not stated	In mothers of pre-school children, the perceived barriers that enable or inhibit them to ensure their children are physically active in their leisure time	*n* = 54 (100% female)	No other demographic details supplied	Aged 4 years	**
Roscoe et al. (2017) [41]	Phenomenological via semi-structured interviews (purposive sample)	UK: North Warwickshire Area of high deprivation		Pre-school staff and parents’ perceptions of pre-school children’s PA and FMS, considering the environment, facilities, play, socio-economic status, and PA barriers	*n* = 10 (100% female)	Unable to separate any other details from those of staff also included in focus groups	Aged 2–4 years	*****
Stirrup et al. (2015) [42]	Mixed methods Ethnography via semi-structured interviews (part of a larger, ongoing study—only qualitative data relevant; purposive sample)	UK: Single unspecified local authority	Parent of child aged 0–5 years; attending pre-school facility; classified as middle class	Opportunities for pre-school children (aged 0–5 years) to participate in PA, the influence of social class and culture on parents’ opportunities (and dispositions) to invest in and their choice of PA experiences. Plus, via Bourdieu’s concepts of habitus and capital, the tendency for parents to provide copious developmental opportunities via ‘intensive mothering’	*n* = 3 interviews (100% female; all selected from sample of *n* = 79 questionnaire respondents as examples of middle-class)	No demographic details supplied for interview participants	Aged 0–5 years	***

^a^ σ = standard deviation; ECC = early childcare centre; FMS = fundamental movement skills; *n* = number; PA = physical activity; SES = socioeconomic status; TAFE = technical and further education; UK = United Kingdom; USA = United States of America. ^b^ Overall study quality was assessed using the Mixed Methods Assessment Tool (MMAT) and is reported using asterisks (*) as a descriptor, ranging from 1*, where 20% of the quality criteria have been met, to 5*, where 100% of the quality criteria have been met [14].

## Data Availability

The data that support the findings of this study are available from the corresponding author upon reasonable request.

## Supplementary Materials

The following supporting information can be downloaded at: https://www.mdpi.com/article/10.3390/children9111727/s1, Table S1: Coding criteria for the Mixed Methods Assessment Tool; Table S2: Quality assessment results from Mixed Methods Assessment Tool; File S1: Database-specific terms, restrictions and search strategies, and secondary and grey literature search processes; File S2: Themes and indicative text quotes.
