# Supplementary material for: Mixed-Methods Systematic Review to Identify Facilitators and Barriers for Parents/Carers to Engage Pre-School Children in Community-Based Opportunities to Be Physically Active
Source: Children (Basel). 2022 Nov 10;9(11):1727. doi: 10.3390/children9111727 (PMC9688361; doi:10.3390/children9111727)
Supplement: Supplementary file 1 [file children-09-01727-s001.zip › children-2002427-supplementary1.pdf]

children MDPI

Electronic Supplementary Material 1 Database-specific terms, restrictions and search strategies, and secondary and grey literature search processes

Database Specific Restrictions

- Publication restrictions – separate searches were run in each database for Academic Journals (peer reviewed) and grey literature to allow the separate documentation of retrieved studies. One search was restricted to only Academic Journals only (peer-reviewed), the other excluded Academic journals, and use database specific publication restrictions as detailed below.
- MeSH terms and keyword searches were explored during preliminary searches. Due to the quality of citations generated and number of specific terms not covered by MeSH, keyword not MeSH term searches were subsequently run within Medline and CINHAL.

Bibliographic Database Specific Search Strategies – All run 16th May 2022

Web of Science

https://www.webofscience.com/wos/woscc/summary/689385a0-7843-4cf0-a9b8-97278eef7fdd-3860f1a3/relevance/1

#1	((((((((((((((TI=(parent*)) OR AB=(parent*)) OR TI=(carer*)) OR AB=(carer*)) OR TI=(grandparent*)) OR AB=(grandparent*)) OR TI=(guardian*)) OR AB=(guardian*)) OR TI=(mother*)) OR AB=(mother*)) OR TI=(father*)) OR AB=(father*)) OR TI=(grandfather*)) OR AB=(grandfather*)) OR TI=(grandmother*)) OR AB=(grandmother*)
#2	((((((((((((((((((TI=(preschool*)) OR AB=(preschool*)) OR TI=(pre-school*)) OR AB=(pre-school*)) OR TI=("preschool-aged")) OR AB=(preschool-aged)) OR TI=(kindergarten)) OR TI=("early years")) OR AB=("early years")) OR AB=(kindergarten)) OR TI=(" early childhood")) OR AB=("early childhood")) OR TI=("young children")) OR AB=("young children")) OR TI=(infants)) OR AB=(infants)) OR TI=(toddlers)) OR AB=(toddlers)) OR TI=(babies)) OR AB=(babies)
#3	((TI=(barrier*)) OR AB=(barrier*)) OR TI=(facilitator*)) OR AB=(facilitator*)) OR TI=(enabl*)) OR AB=(enabl*)) OR TI=(assist*)) OR AB=(aassist*)) OR TI=(motivat*)) OR AB=(motivat*)) OR TI=(support*)) OR AB=(support*)) OR TI=(determin*)) OR AB=(determin*)) OR TI=(factors)) OR AB=(factors)) OR TI=(predict*)) OR AB=(predict*)) OR TI=(increas*)) OR AB=(increas*)) OR AB=(decreas*)) OR TI=(decreas*)) OR TI=(influenc*)) OR AB=(influenc*)) OR TI=(encourage*)) OR AB=(encourage*)) OR TI=(correlat*)) OR AB=(correlat*)) OR TI=(attitude*)) OR AB=(attitude*)) OR TI=(perception*)) OR AB=(perception*)) OR TI=(views)) OR AB=(views)) OR TI=(reasons)) OR AB=(reasons)) OR TI=(opportunities)) OR AB=(opportunities)) OR TI=(opinions)) OR AB=(opinions)) OR TI=(challeng*)) OR AB=(challeng*)) OR TI=(difficult*)) OR AB=(difficult*)) OR AB=(prevent*)) OR TI=(prevent*)) OR TI=(constraint*)) OR AB=(constraint*)) OR TI=(limit*)) OR AB=(limit*)
#4	((((((((((((((((((((((((TI=("physical activit*")) OR AB=("physical activit*")) OR TI=(playgroup)) OR AB=(playgroup)) OR TI=("free play")) OR AB=("free play")) OR TI=(free-play)) OR AB=(free-play)) OR TI=("active play")) OR AB=("active play")) OR TI=("fundamental movement")) OR AB=("fundamental movement")) OR TI=(exercise)) OR AB=(exercise)) OR TI=(sport)) OR AB=(sport)) OR TI=("motor skills")) OR

	AB=("motor skills")) OR TI=(recreation)) OR AB=(recreation)) OR TI=(" motor competenc*")) OR AB=("motor competenc*")) OR TI=(" tummy time")) OR AB=("tummy time")) OR TI=("physical literacy")) OR AB=("physical literacy")
#5	#1 AND #2 AND #3 AND #4
#6	**#1 AND #2 AND #3 AND #4** and **2022** or **2021** or **2020** or **2019** or **2018** or **2017** or **2016** or **2015** (Publication Years)
#7	**#1 AND #2 AND #3 AND #4** and **2022** or **2021** or **2020** or **2019** or **2018** or **2017** or **2016** or **2015** (Publication Years) and **Articles** (Document Types)
#8	**#1 AND #2 AND #3 AND #4** and **2022** or **2021** or **2020** or **2019** or **2018** or **2017** or **2016** or **2015** (Publication Years) and **English** (Languages) and **Articles** or **Early Access** (Document Types)

ProQuest and ASSIA via ProQuest Central

S1	(ti(parent*) OR ab(parent*) OR ti(carer*) OR ab(carer*) OR ti(grandparent*) OR ab(grandparent*) OR ti(guardian*) OR ab(guardian*) OR ti(mother*) OR ab(mother*)
S2	(ti(father*) OR ab(father*) OR ti(grandfather*) OR ab(grandfather*) OR ti(grandmother*) OR ab(grandmother*)
S3	(ti(preschool*) OR ab(preschool*) OR ti(pre-school*) OR ab(pre-school*) OR ti(preschool-aged) OR ab(preschool-aged) OR ti(kindergarten) OR ab(kindergarten) OR ti("early years") OR ab("early years")
S4	(ti("early childhood") OR ab("early childhood") OR ti("young children") OR ab("young children") OR ti(infants) OR ab(infants) OR ti(toddlers) OR ab(toddlers) OR ti(babies) OR ab(babies)
S5	(ti(barrier*) OR ab(barrier*) OR ti(facilitator*) OR ab(facilitator*) OR ti(enabl*) OR ab(enabl*) OR ti(assist*) OR ab(assist*) OR ti(motivat*) OR ab(motivat*)
S6	(ti(support*) OR ab(support*) OR ti(determin*) OR ab(determin*) OR ti(factors) OR ab(factors) OR ti(predict*) OR ab(predict*) OR ti(increas*) OR ab(increas*)
S7	(ti(decreas*) OR ab(decreas*) OR ti(influenc*) OR ab(influenc*) OR ti(encourage*) OR ab(encourage*) OR ti(correlat*) OR ab(correlat*) OR ti(attitude*) OR ab(attitude*)
S8	(ti(perception*) OR ab(perception*) OR ti(views) OR ab(views) OR ti(reasons) OR ab(reasons) OR ti(opportunities) OR ab(opportunities) OR ti(opinions) OR ab(opinions)
S9	(ti(challeng*) OR ab(challeng*) OR ti(difficult*) OR ab(difficult*) OR ti(prevent*) OR ab(prevent*) OR ti(constraint*) OR ab(constraint*) OR ti(limit*) OR ab(limit*)
S10	(ti("physical activit*") OR ab("physical activit*") OR ti(playgroup*) OR ab(playgroup*) OR ti("free play") OR ab("free play") OR ti("free-play") OR ab("free-play") OR ti("active play") OR ab("active play")
S11	(ti("fundamental movement") OR ab("fundamental movement") OR ti(exercise) OR ab(exercise) OR ti(sport) OR ab(sport) OR ti("motor skills") OR ab("motor skills") OR ti(recreation) OR ab(recreation)
S12	(ti("motor competenc*") OR ab("motor competenc*") OR ti("lifestyle choices") OR ab("lifestyle choices") OR ti("tummy time") OR ab("tummy time") OR ti("physical literacy") OR ab("physical literacy")
S13	S1 OR S2
S14	S3 OR S4
S15	S5 OR S6 OR S7 OR S8 OR S9
S16	S10 0R S11 0R S12
S17	S13 AND S14 AND S15 AND S16
Limits	(at.exact("Article") AND stype.exact("Scholarly Journals") AND la.exact("ENG") AND pd(20150101-20221231) AND PEER(yes))

EBSCOhost SPORTDiscus and EBSCOhost CINHAL

S1 TI parent* OR AB parent* OR TI carer* OR AB carer* OR TI grandparent* OR AB grandparent* OR TI guardian* OR AB guardian* OR TI mother* OR AB mother* OR TI father* OR AB father* OR TI grandfather* OR AB grandfather* OR TI grandmother* OR AB grandmother*

S2 TI preschool* OR AB preschool* OR TI pre-school* OR AB pre-school* OR TI preschool-aged OR AB preschool-aged OR TI kindergarten OR AB kindergarten OR TI "early years" OR AB "early years" OR TI "early childhood" OR AB "early childhood" OR TI "young children" OR AB "young children" OR TI infants OR AB infants OR TI toddlers OR AB toddlers OR TI babies OR AB babies

S3 TI barrier* OR AB barrier* OR TI facilitator* OR AB facilitator* OR TI enabl* OR AB enabl* OR TI assist* OR AB assist* OR TI motivat* OR AB motivat* OR TI support* OR AB support* OR TI determin* OR AB determin* OR TI factors OR AB factors OR TI predict* OR AB predict* OR TI increas* OR AB increas* OR TI decreas* AB decreas* OR TI influenc* OR AB influenc* OR TI encourage* OR AB encourage* OR TI correlat* OR AB correlat* OR TI attitude* OR AB attitude* OR TI perception* OR AB perception* OR TI views OR AB views OR TI reasons OR AB reasons OR TI opportunities OR AB opportunities OR TI opinions OR AB opinions OR TI challeng* OR AB challeng* OR TI difficult* OR AB difficult* OR TI prevent* OR AB prevent* OR TI constraint* OR AB constraint* OR TI limit* OR AB limit*

S4 TI "physical activit*" OR AB "physical activit*" OR TI playgroup* OR AB "playgroup*" OR TI “free play” OR AB “free play” OR TI “free-play” OR AB “free-play” OR TI “ active play” OR AB “active play” OR TI “fundamental movement” OR AB “fundamental movement” OR TI exercise OR AB exercise OR TI sport OR AB sport OR TI “motor skills” OR AB “ motor skills” OR TI recreation OR AB recreation OR TI “motor competenc*” OR AB “motor competenc*” OR TI “lifestyle choices” OR AB “lifestyle choices” OR TI “tummy time” OR AB “tummy time” OR TI “physical literacy” OR AB “ physical literacy”

S5 S1 AND S2 AND S3 AND S4

Expanders - Apply equivalent subjects

Search modes - Boolean/Phrase

Limiters - Published Date: 20150101-20221231; Publication Type: Academic Journal

EBSCOhost MEDLINE

S1 TI parent* OR AB parent* OR TI carer* OR AB carer* OR TI grandparent* OR AB grandparent* OR TI guardian* OR AB guardian* OR TI mother* OR AB mother* OR TI father* OR AB father* OR TI grandfather* OR AB grandfather* OR TI grandmother* OR AB grandmother*

S2 TI preschool* OR AB preschool* OR TI pre-school* OR AB pre-school* OR TI preschool-aged OR AB preschool-aged OR TI kindergarten OR AB kindergarten OR TI "early years" OR AB "early years" OR TI "early childhood" OR AB "early childhood" OR TI "young children" OR AB "young children" OR TI infants OR AB infants OR TI toddlers OR AB toddlers OR TI babies OR AB babies

S3 TI barrier* OR AB barrier* OR TI facilitator* OR AB facilitator* OR TI enabl* OR AB enabl* OR TI assist* OR AB assist* OR TI motivat* OR AB motivat* OR TI support* OR AB support* OR TI determin* OR AB determin* OR TI factors OR AB factors OR TI predict* OR AB predict* OR TI increas* OR AB increas* OR TI decreas* AB decreas* OR TI influenc* OR AB influenc* OR TI encourage* OR AB encourage* OR TI correlat* OR AB correlat* OR TI attitude* OR AB attitude* OR TI perception* OR AB perception* OR TI views OR AB views OR TI reasons OR AB reasons OR TI opportunities OR AB opportunities OR TI opinions OR AB opinions OR TI challeng* OR

AB challeng* OR TI difficult* OR AB difficult* OR TI prevent* OR AB prevent* OR TI constraint* OR AB constraint* OR TI limit* OR AB limit*

S4 TI "physical activit*" OR AB "physical activit*" OR TI playgroup* OR AB "playgroup*" OR TI “free play” OR AB “free play” OR TI “free-play” OR AB “free-play” OR TI “ active play” OR AB “active play” OR TI “fundamental movement” OR AB “fundamental movement” OR TI exercise OR AB exercise OR TI sport OR AB sport OR TI “motor skills” OR AB “ motor skills” OR TI recreation OR AB recreation OR TI “motor competenc*” OR AB “motor competenc*” OR TI “lifestyle choices” OR AB “lifestyle choices” OR TI “tummy time” OR AB “tummy time” OR TI “physical literacy” OR AB “ physical literacy”

S5 S1 AND S2 AND S3 AND S4

Limiters - Scholarly (Peer Reviewed) Journals; Date of Publication: 20150101-20221231

Expanders - Apply equivalent subjects

Search modes - Boolean/Phrase

Secondary and Grey Literature Searching

- Google Advance Search and an identified list of topic specific websites were searched for grey literature; full text articles retrieved were hand searched via reference checking and forward and backwards citation screening/snowballing, and members of the wider review team from Sport Wales and Local Councils in Wales were contacted to identify any additional studies.

Google Advanced Search

Tuesday 31st May 12:20pm

All of these words: physical activity

Any of these words: "early years", "young children", preschool, "physical literacy", "early childhood", "active play", sport, exercise, toddlers, infants

Limiters

Language: English

Search run with and without limiting *region* to: “United Kingdom”
