# Supplementary material for: Mixed-Methods Systematic Review to Identify Facilitators and Barriers for Parents/Carers to Engage Pre-School Children in Community-Based Opportunities to Be Physically Active
Source: Children (Basel). 2022 Nov 10;9(11):1727. doi: 10.3390/children9111727 (PMC9688361; doi:10.3390/children9111727)
Supplement: Supplementary file 1 [file children-09-01727-s001.zip › children-2002427-supplementary2.pdf]

children

MDPI

Electronic Supplementary Material Breakdown of the quality assessments and supporting justification

Table S1. Coding criteria for the Mixed Methods Assessment Tool.

**Code Breakdown**	
Screening questions (For all types, if no, assessment should not continue)	S1. Are there clear research questions?
	S2. Do the collected data allow to address the research questions?
1. Qualitative	1.1. Is the qualitative approach appropriate to answer the research question?
	1.2. Are the qualitative data collection methods adequate to address the research question?
	1.3. Are the findings adequately derived from the data?
	1.4. Is the interpretation of results sufficiently substantiated by data?
	1.5. Is there coherence between qualitative data sources, collection, analysis and interpretation?
2. Quantitative randomized controlled trials	2.1. Is randomization appropriately performed?
	2.2. Are the groups comparable at baseline?
	2.3. Are there complete outcome data?
	2.4. Are outcome assessors blinded to the intervention provided?
	2.5 Did the participants adhere to the assigned intervention?
3. Quantitative non-randomized	3.1. Are the participants representative of the target population?
	3.2. Are measurements appropriate regarding both the outcome and intervention (or exposure)?
	3.3. Are there complete outcome data?
	3.4. Are the confounders accounted for in the design and analysis?
	3.5. During the study period, is the intervention administered (or exposure occurred) as intended?
4. Quantitative descriptive	4.1. Is the sampling strategy relevant to address the research question?
	4.2. Is the sample representative of the target population?
	4.3. Are the measurements appropriate?

	4.4. Is the risk of nonresponse bias low?
	4.5. Is the statistical analysis appropriate to answer the research question?
5. Mixed methods	5.1. Is there an adequate rationale for using a mixed methods design to address the research question?
	5.2. Are the different components of the study effectively integrated to answer the research question?
	5.3. Are the outputs of the integration of qualitative and quantitative components adequately interpreted?
	5.4. Are divergences and inconsistencies between quantitative and qualitative results adequately addressed?
	5.5. Do the different components of the study adhere to the quality criteria of each tradition of the methods involved?

Table S2. Quality assessment results from Mixed Methods Assessment Tool.

	**1. Qualitative Studies**					**2. Randomized Controlled Trials**					**3. Non-Randomized Studies**					**4. Quantitative Descriptive Studies**					**5. Mixed Methods Studies**						
	1.1.	1.2.	1.3.	1.4.	1.5.	2.1.	2.2.	2.3.	2.4.	2.5.	3.1.	3.2.	3.3.	3.4.	3.5.	4.1.	4.2.	4.3.	4.4.	4.5.	5.1.	5.2.	5.3.	5.4.	5.5.	**Overall Score**	**Comments**
Allen et al. (2021)	Y	Y	Y	Y	U																					4	Difficult to tell for 1.4 and 1.5 because the results and discussion are combined, therefore data appears to be minimal, but there are quotes to support findings
Andrews et al. (2019)	Y	Y	U	U	U											Y	N	Y	N	Y	N	Y	Y	Y	N	2	1.3, 1.4, 1.5 no details of thematic analysis approach, data collection, analysis or how themes derived. Quantitative & Qualitative data integrated. 4.3 unvalidated questionnaire but trialled prior to use. 4.4 risk of

																											non-response bias from outer suburbs 5.1 no rationale for mixed method approach. 5.5 no information on quality /trustworthiness processes 5.4 no apparent divergences
Downing et al. (2016)	Y	Y	U	Y	Y																					4	1.3 lack of details on processes of thematic analysis, and trustworthiness of data analysis. Only qualitative data relevant.
Fuller et al. (2019)	Y	Y	Y	Y	Y																					5	No identifiable issues
Grindley (2022)	Y	Y	Y	Y	Y																					5	No identifiable issues
Grzywacz et al. (2016)	Y	Y	Y	Y	Y																					5	No identifiable issues
Hnatiuk et al. (2020)	Y	Y	Y	Y	Y																					5	No identifiable issues
Houghton et al. (2015)	Y	Y	Y	Y	Y																					5	No identifiable issues
Joseph et al. (2019)	Y	Y	Y	Y	Y																					5	No identifiable issues

Lindsey et al. (2022)	Y	Y	Y	Y	Y																					5	No identifiable issues
Lindsey et al. (2019)	Y	Y	Y	Y	Y																					5	No identifiable issues
Martin-Biggers et al. (2015)	Y	Y	Y	Y	Y																					5	No identifiable issues. Only qualitative data relevant.
Penilla et al. (2017)	Y	Y	Y	Y	Y																					5	No identifiable issues
Pisot (2020)	Y	Y	U	U	U																					2	1.3, 1.4, 1.5 No details on qualitative data analysis or interpretation. mixed method but only qualitative data relevant
Roscoe et al. (2017)	Y	Y	Y	Y	Y																					5	No identifiable issues
Stirrup et al. (2015)	Y	Y	Y	U	U																					3	Difficult to assess as the methodology definition is unclear. Authors themselves state that data is sparse

Y = yes; N = no; U = unclear.
