# Supplementary material for: Mixed-Methods Systematic Review to Identify Facilitators and Barriers for Parents/Carers to Engage Pre-School Children in Community-Based Opportunities to Be Physically Active
Source: Children (Basel). 2022 Nov 10;9(11):1727. doi: 10.3390/children9111727 (PMC9688361; doi:10.3390/children9111727)
Supplement: Supplementary file 1 [file children-09-01727-s001.zip › File S2.pdf]

children

MDPI

Electronic Supplementary Material File 2: Themes and Indicative text quotes

SEM theme	Main theme	Sub themes		
Individual	*Beliefs*	**Fostering broad child development traits**	Perception that the culture of sport fosters child development skills - team work	*'It wasn't particularly that we were drawn to football it was just that we wanted him to do something else that was sport related, and particularly sort of more team centred so that he sort of develops those skills of working in a team as well as just being a sporting activity'*. **(1)**
			Participation as a way for child to gain an essential life skill (personal safety)	*'Water's everywhere. There's a risk in a lot of things you do around water, they have to be able to swim to do lots of other things in life'* **(1)**<br><br>*'I think swimming's a life skill that you need to have.'* **(1)**
			Attending bouldering as a way to develop social and emotional skills	The importance of learning from others and acquiring a skill that could be used to enhance family bonds were both cited as reasons for initially attending the classes<br>*We thought Rock Tots would be good because he would be interacting with the other kids, learning from people who know how to teach young children how to climb, and then playing other games that are beneficial other than just getting on the wall and having a go i suppose.* (P4, Father) **(6)**<br><br>All parents recognised the potential benefits of the classes on their children's developing social skills. **(6)**

			Perceived potential exposure to additional stimulating learning opportunities and environments as a facilitator	Most parents indicated their desire to seek out opportunities that facilitated the generation of relevant academic capital, i.e., they sought environments that provided 'positive learning opportunities' **(16)**<br><br>Activities are also valued because of a belief they help to develop a range of skills and include socialisation<br>: *"We go to the local playgroup in the village once a week. We like to go for walks sometimes we will take the balance bikes with us. We do a craft class and we do cake building classes and we go to Lego Club as well. We've done Little Kickers football which he loved. We've done the balance bike classes; he loves to run or play football. What else have we done? Trampolining and Baby Gym is really good as well, so we've been to that quite a bit"* (Parent 5). **(1)**
			Perceiving PA as a way to develop confidence as a facilitator	Other' reasons given included improving confidence, **(16)**
			Participation (in private swimming lessons) provides the child with early childhood education for long-term advantage	*'We wanted him to learn to swim for when he's at school. If he does start lessons at school, he'll already know what he's doing.'* **(1)**
			Attending the park for social skill development and companionship	*'There's always people at the park because it's such a busy area with so many kids his age, you'll always see people there'***(2)**
			PA providing opportunity for children to develop social and emotional skills as a facilitator	'providing positive social experiences (for children and parents) and presenting opportunities for children to be active or exercised. **(16)**

			Recognition that other social skills can be learnt and developed through participation in structured sporting activities (social skills and listening to others)	As Parent 2 notes, they are aware of the importance of developing skills that can be taught through socialising in a sporting structured activity: *'in terms of social skills y'know I could see that she was chatting to other children'*. Other skills that are valued are listening to others: *'to learn to listen to the teacher and do as she's told'* (Parent 2). Developing the ability to listen to others and socialise are key skills which help support children in their transition into school and spaces that facilitate learning. **(1)**
**Individual**	***Beliefs***	**Good Parenting**	Mitigating safety worries as facilitator to enrolment in swimming lessons	Most of the parents used words such as 'safety', 'safe', 'fear', and 'danger' to explain why they took their infants swimming, reflecting a fear that many parents have with regards to water safety and drowning **(1)**<br><br>Water Babies were popular with parents she knew and that advertising via social media was an effective way of ensuring that parents were willing to spend money on these classes as such advertising played on fear and offered a way to mitigate that through consuming classes. **(1)**
			Enrolment in swimming lessons as wanting to comply with social norms	Parents in this study also discussed class habitus and the extensive marketing of such activities as 'good' parenting in order to keep children safe as a reason for participating in such classes. Parent 6 started organised swimming classes when her little girl was just a baby:<br>*'Right from being a baby she went to Water Babies for two terms, and then she started group lessons and one- to-one lessons. I knew lots of people who took their little ones to Water Babies, I'd seen adverts on Facebook and in the mother-to-be packs that you get, and things like that that were advertised quite heavily.'* **(1)**

**Individual**	***Beliefs***	**Benefits of professional instruction**	Perception that paying for skilled instructor /appropriate role model will maximise child's learning and behaviour, facilitated enrolment in private swimming lessons	*'And I think potentially because I don't have that confidence she picks up on that, I have tried a couple of times recently when I've taken her swimming to encourage her to take her arm bands off but she doesn't seem to feel as comfortable with me doing it so I thought if she if she goes in with someone who knows what they're doing then she's going to pick up on that and trust what they're saying. I think she's more likely to listen to someone else probably than listen to me and follow the rules'* **(1)**
			Organised class with professional present, facilitator of attendance for fathers	Fathers were also more likely to attend if CC Staff were present during the sessions. **(8)**
			Perception that individual-approach provides greater gains than group-based approach	*I think perhaps with the groups there's too many children in [the pool] and I think that they just sit on the side of the pool for the most of it and maybe then he gets distracted and mess' around, which they're going to at that age. So, I think the one-to-ones are brilliant and I think they learn so much more than they do in a group lesson. I think it's probably quite an expensive weekly activity, but I just think that we've done the groups and I think it's worth it. I think that we get so much more out of it. It's worth the commitment. I think perhaps the one-to-ones are expensive, but you get so much more out of a one-to-one.* **(1)**
			Anticipated development of strong sport-specific foundational skills from a professional instruction a motivation for attendance at bouldering classes	Other parents indicated that the anticipated benefits of their children receiving sport specific instruction was the main motivator for attending the classes<br>*One of the main motivations for doing Rock Tots was that whilst I climb I've never been taught how to do it but I thought it would be really useful for them to kind of learn it properly rather than just like playing around and doing it. But it's really useful for them to have some kind of guided instruction as well so that was the motivation for doing it really* (P3, Mother) **(6)**

**Individual**	***Knowledge***	**Prior Knowledge**	Recognition that early physical skill development beneficial for securing leisure opportunities in later life	Parents stressed how physical skills such as good co-ordination were worth investing in. Through investing in early years activities parents recognise that this can secure their children access to a range of leisure opportunities both now and in the future. This is highlighted by parent 6 who explains, *'other than swimming she goes to gymnastics once a week and that's a group gymnastics session just to try and help her balance and co-ordination'*. **(1)**
			PA helps prevent childhood obesity	Parents also said that they wanted structural changes such as more clean and safe parks in order to prevent childhood obesity.
			Perception of health and fitness benefits of attending bouldering sessions	Some parents initially reported the health and fitness elements that brought them to the sessions. *We felt it was a really good all round fitness and there's something that you can do from like any age group up to any age group you know, you see a lot different age groups of people in the indoor climbing wall then there's all the fitness.* ***(6)***
	Knowledge	**Lack of Knowledge**	Fathers' perception that toddler groups are mainly for mothers a barrier to attendance (gender dominance)	perceptions that the CC was perceived as a female environment. In respect of the latter one father commented: … Often when you go to normal toddler groups it's mostly mums and there's hardly any dads F65. **(8)**
			Not having the skills to teach their child themselves facilitated attendance at private swimming lessons	During the interviews, participants were asked to discuss why they selected structured and organised swimming lessons for their toddlers. One of the key themes was that parents felt they lacked knowledge, as Parent 2 highlights, paying for professionals to teach swimming skills seems to be part of ensuring the development of physical capital: *'I have taken her on my own but I'm not skilled enough to teach her how to swim.'* **(1)**
			Lack of understanding about the sessions a barrier to attendance at classes (for fathers)	lack of understanding about the sessions and how this was conveyed through advertising; **(8)**

**Individual**	***Parental Parameters***		Health of the parent	Barriers to engagement were linked to external variables and circumstances such as illness or disability of parent and/or child; **(8)**
			Personality traits of fathers as a barrier to participation	parental laziness, shyness, and (particularly work schedules); **(8)**
**Interpersonal /Social Environment**	***Social Benefits***	**Parental**	Social benefits for the parent facilitate attending playgroup	*'We're a new estate here... Ah, yeah and I'm also on a lot of committees and stuff so I meet people through a lot of avenues... We also run a playgroup in the estate, so we meet a lot of people socially through that and he goes to that as well.'* (P77 outer) **(2)**
			Attending the park as an opportunity for parents (mothers) to socialise	*I sort of say, "Oh let's go to the park" because the kids are getting exercise, they're running around at the same time as me socialising, so it's sort of, it works for me on both fronts, whereas I just find that that doesn't tend to come into their decision- making as much as it would mine.* **(7)**
			PA as a social opportunity for parents as a facilitator	. 'providing positive social experiences (for……..parents) and presenting opportunities for children to be active or exercised. **(16)**
			Opportunity to socialise as a facilitator of park attendance for mothers	Having a friend [mother] who also has children about the same age make it easier for everyone . . .the kids play and we [mothers] talk. . .It's just makes it better to have company and you feel like getting out with the kids more. . . FGD #3; Mother #17**(11)**

	Family Dynamic	**Sibling age differences**	Competing child commitments is a barrier to PA	Some parents described how challenges arose for families when their children were at particular or varying ages and stages of life (e.g., they had a young baby at home or a child who was quite a bit older than their 2- to 4-year-old child) because they had to juggle multiple commitments (e.g. sporting practice for an older child) **(7)**
			Having children of different ages, outside of class age range a barrier to participation (for fathers)	needing to engage multiple children within/outside the programme target age range; **(8)**
**Interpersonal /Social Environment**	***Family Dynamic***	**Role of the mother**	Mothers perceive that they drive children's engagement/enrolment in new PA opportunities	From the point of family support to child's PA, interviews showed that mothers are the main motivators (2/3) for a child to learn a new sport (rollerblading, cycling, skiing, swimming), **(14)**<br><br>*"... Otherwise, the initiative comes from me because I spend more time with the children,* **(14)**
			Mothers delegate teaching of new sports to fathers or professionals	they leave the teaching of new sports to the fathers (60%) or professionals. **(14)**
			Fathers perceive mothers to be the drivers/ parental role within family dynamic as a facilitator/barrier to PA	A number of fathers also made reference to the role of the mother as a gatekeeper and organiser suggesting that they would not have attended the sessions or been aware of the programme if they had not been encouraged to attend by the child's mother. For example:<br>... I suppose for me, how do I put this? My wife's the organiser ... .if there's a Dad's club or something sort of going on (she will say) why don't you go along there and that's what we've done. F32**(8)**

**Interpersonal /Social Environment**	***Social Network***	**Peer influence**	Peer influence facilitating decisions to attend private swimming lessons	it was essential to go to baby swimming because close friends and '*everyone else*' they knew were taking their children: '*We'd heard a lot of the other kids were doing it [swimming] and my wife's friend's son, well he'd been doing it pretty much since he was born, so we just thought everyone else is [taking their children] so we might as well try as well.*' **(1)**
			Social networks as a barrier to PA	when local networks of parents held differing attitudes towards physical activity, it often served as a barrier to co-participation in physical activity, as described below: *. . . they'd be like, "Let's go meet at the cafe´" and we'll sit there and, you know, have a biscuit and coffee, and it's all very nice—I enjoy it, but for me, I try to limit the occasions that we do that* **(7)**
**Interpersonal /Social Environment**	***Social Network***	**Parental Peer Support**	Feeling of togetherness as a facilitator of attending playgroups	the support received from their playgroup peers, including older parents or grand-parents attending the playgroup, and an attitude that "we are all in it together**". (4)**
			Comradeship and ability to share parenting practices in a safe space a reason for attending playgroups	receiving support from their peers was identified as a major reason for attending playgroup. "I think also sharing stories, talking to other mums and sharing what's happened during the week, and then going hey, you're not the only one." Parent, FG1. "What we all bring is different experiences and different ways of doing things, so you can talk to someone about what they do and then that might work for you and someone else might have something different to offer, so that's what's good about a group environment," Parent, FG4. **(4)**

			Opportunity for parent-child observations to inform one's own future parenting practices	The mothers also talked about the benefits of being able to observe other parents interacting with their child at playgroup. This included observing older children, to gain an insight into what to expect when their own child reached that developmental stage. "I struggled a lot for a long time, but the supportive network at playgroup was good, where you looked to everyone for ideas and different approaches." Parent, FG4. **(4)**
			Social networks and safe environments facilitate use of parks	importance of social networks and neighbourhood safety as factors that influenced physical activity co-participation. For instance, parents spoke of how their children's friends positively influenced their family's physical activity participation, as they often met other families who live locally at parks and/or used social media to connect to events in their community: *I have a couple of friends that have children around the same age . . . so, we'll all go out to the park together as a family. There's a big reserve near where I live and they've got big pathways and water features. So, we'll all take the kids out there, go for a walk and let the kids run around and play. (Jane)* **(7)**
			Sharing knowledge between peers raises awareness of PA opportunities for mothers	some mothers spoke of their network of friends (e.g., other Brazilian parents) positively influencing their children's PA by their provision of informational and/or logistic support. For example, mothers mentioned that having friends tell them about PA classes and organized sports classes, motivated them to enrol their own children in similar activities **(11)**
			For mothers, good social networks enable sharing of resources between families to facilitate child's attendance at organised sports/ PA	My next-door neighbour and me take turns taking the kids to swim lessons. It's really helpful because sometimes I need to get home later from work and we help each other that way. . . FGD #6; Mother #27 **(11)**

			Established social networks with other parents as a facilitator park attendance for mothers	mothers reported that having close friends with young children motivated them to get together and take their children to parks and playgrounds. **(11)**
**Built & Physical Environment**	***Infrastructure***	**Parks & Open Space**	Access to open space was a greater hinderance to play in outer suburbs than inter suburbs	Access to open space 4 (5.6%) inner, 4 (15.4%) outer. **(2)**
			Access to more outdoor space and equipment facilitates going to the park	*'……or for swings which he loves, which we don't have so we'll go there for that... or to kick a ball because we don't have room for that'. (P55 inner)***(2)**
			A lack of resources (Parks and green spaces) for safe play as a barrier to PA for fathers	Some fathers also mentioned a lack of neighbourhood resources such as parks and green spaces where children and families could play safely **(10)**
			Lack of parks in rural locations a barrier to play/PA	Access to parks in this predominantly rural region was virtually non-existent. **(5)**
			Well-resourced parks with age-appropriate equipment and all-weather provisions	good access to local parks/playgrounds with equipment suitable for young children and shaded areas, as well as open spaces **(7)**
			Access to outdoor facilities as a barrier to play in urban areas	you feel a bit. . . you can only go to play centres so often and you want to make it... What else can I do with the kids that's outside? (Urban, high educated mother) **(3)**
**Built & Physical Environment**	***Infrastructure***	**Accessibility of-Resources**	Accessibility of indoor play facilities a barrier to play	Some mothers reported that they often looked for new play or activity ideas due to limited space at home or poor accessibility to indoor play areas.<br>Yeah, there's a couple of sites that I've looked at because sometimes... well, especially in winter when it's raining and we've got a fairly small house and **(3)**

			Accessibility of 'all weather' facilities in rural areas a barrier to play	Like up there they have play centres and stuff they don't have down here, so you're limited to perhaps finding a park with the weather permitting or. . . that's why I did go online to try and find activities to do at home. (Regional, high educated mother) **(3)**
			Ability to access suitable services and programmes influences PA	key social environment factors influencing physical activity for their children included access to services and programmes, such as playgroups, childcare and local council events**(7)**
**Built & Physical Environment**	***Infrastructure***	**Quality of Resources**	Quality of public spaces more or a barrier to play in outer suburbs	Quality of public space 4 (5.6%) inner, 8 (30.8%) outer **(2)**
			Quality of facilities as a barrier to going to the park	*We've got one park, it's not very good but it's all that we've got'.* **(2)**
			Deteriorating condition of park equipment as a barrier to attendance	*'equipment that's there is really not that good, there's old equipment that's been broken down and old parts that have been removed so like a slide removed but not replaced'.* (P79 outer) **(2)**
			Broken equipment and safety hazards (broken glass) as a barrier/facilitator of park use	Parents also said that they wanted structural changes such as more clean and safe parks in order to prevent childhood obesity. Parents reported that under-policing and park conditions require additional vigilance if and while their children use a park: for example, *"You see broken glass, you see a basketball, but no rims, the parks are empty...they [parents] just rather keep their kids at home because of gang violence, drugs...they buy them a TV, play station, Xbox, or computer, as long as they don't bother nobody."* **(13)**
**Built & Physical Environment**	***Infrastructure***	**Transportation**	Access to transport only a barrier to play in outer suburbs compared to inner suburbs	Access to transport 0 inner, 4 (15.4%) outer. **(2)**

			Lack of personal transportation a barrier to Childs PA	a further barrier which was identified by parents was 'cost and the ability to drive to get my son places for exercise' **(15)**
			Access to transportation constrained PA in rural farmland areas in USA	Within this context transportation constrained children's physical activity. Few farmworker families had their own mode of transportation; and even when a vehicle was available, many mothers did not have a valid drivers' license, or they feared driving over concerns of police checkpoints. **(5)**
**Built & Physical Environment**	***Infrastructure***	**Distance**	Accessibility of facilities in rural areas a barrier to play	I think for me living where I'm living, it's different to the city because in the city there's so many places you can access to take your kids whereas down here it's like. . . what do I do, there's not a lot. **(3)**
			Distance to parks a barrier to PA in rural areas	I haven't seen a park around here. Well, there is one in Wilmington, but it's too far to take them in the afternoon. It takes about an hour to go and come back. **(5)**<br><br>And, sometimes, I don't have time because I only drive to the small towns. I don't drive any further than that to be cautious.<br>Seasonal Family (P#1) **(5)**
			Cost associated with the distance to travel to the nearest park as a barrier to Childs PA	indoor recreation centers (e.g., skating rinks) were expensive, as was the price of transportation ("too much gas because the park is far"). **(12)**
			Distance to parks/local facilities a barrier to PA	parents described how access to local facilities (e.g. living in close proximity to shops and parks), and walk- ability around the neighbourhood created opportunities for physical activity **(7)**

			Distance to parks and green spaces a barrier to PA of fathers	Some fathers also mentioned a lack of neighbourhood resources such as parks and green spaces where children and families could play safely and in close proximity to where they lived as being a barrier to their preschool-age children being physically active. **(10)**<br><br>*Where we live we don't have a playground or a park to take the kids that is near. So, we need drive. We try to take them [sons] to the park as much as we can. It would be easier if we had a playground near the house...* **(10)**
**Built & Physical Environment**	***Infrastructure***	**Safety Concerns**	Concerns about safety were a barrier to play more in outer suburb areas	Concerns about safety 7 (9.7%) inner, 11(42.3%) outer **(2)**
			Location of park and traffic safety concerns as barriers to going to the park	*'Yeah it's sort of sandwiched between a street and two courts and there's heaps of traffic that goes past'***(2)**
			Traffic as a safety issue as a barrier to going to the park in outer suburb areas	Concerns about traffic were a safety issue mentioned by several outer-suburban parents as a barrier to visiting local parks ***(2)***
			Presence of dogs within park areas as a safety concern when using parks in outer suburb areas	in the open-ended interviews, however, traffic wasn't the only safety concern mentioned. One parent explained: *'You've got a certain amount of people they don't care, they just leave their dogs without control, just running around you know, and very often, in certain playgrounds and the football pitch... even when there's kids running around there's still a certain amount of people coming with dogs running all around which is crazy to me'*. (P78 outer) ***(2)***
			Perceived safety (in and surrounding parks) as a barrier to PA	Parents felt that an insufficient number of accessible sidewalks and parks, and a lack of safety measures in existing parks, created a barrier for children's outdoor physical activity. **(9)**

			Fear of anti-social behaviour as a barrier to park use in San Francisco	Parents also said that they wanted structural changes such as more clean and safe parks in order to prevent childhood obesity. Parents reported that under-policing and park conditions require additional vigilance if and while their children use a park: for example, "*You see broken glass, you see a basketball, but no rims, the parks are empty...they [parents] just rather keep their kids at home because of gang violence*
			Safety as a barrier to park use for fathers	*park near our house isn't really good for little kids ...it's not safe* **(10)**
**Community**	***Organisational factors***	**Advertising**	Fear-based marketing techniques facilitate enrolment in private swimming classes	advertising via social media was an effective way of ensuring that parents were willing to spend money on these classes as such advertising played on fear and offered a way to mitigate that through consuming classes. **(1)**
			Social media as a tool to sharing PA ideas and events	*A little bit of social networking . . . if you're in a local community Facebook page or something, people share ideas of what they've done, so even if I don't know the people, you'll see that they've commented that this event is on in the community* **(7)**
			Advertising community PA opportunities would facilitate participation	better advertising of physical activity opportunities would facilitate higher levels of physical activity co-participation **(7)**
			Perceived poor advertising as a barrier to participation	lack of understanding about the sessions and how this was conveyed through advertising; **(8)**
**Community**	***Organisational factors***	**Timing**	Timing of community-based opportunities important	recommended holding more community events (particularly on weekends) **(7)**

			Provision of weekend community events could facilitate PA participation	recommended holding more community events (particularly on weekends) **(7)**
			Class timing as a barrier/facilitator to class attendance (for fathers)	Saturday sessions proved to be the most successful in terms of attendance with a number of fathers indicating that they would have not been able to attend the course if sessions were on another day **(8)**
			Timing of classes a barrier to participation for fathers	session timings clashing with commitments **(8)**
			Class timings as a barrier to mothers enrolling children in PA classes	classes time are not good for my schedule . . . when the kids are older it's easier you can drop them off for soccer and pick them up, but the little ones, you need to stay with them. **(11)**
**Community**	**Organisational factors**	**Target audience**	Developmentally appropriate / unique opportunities based on the principles of learning through play are a facilitator of attendance (bouldering)	All the parents reported that despite the classes being associated with a risky sport, they recognised that the programme had been designed to be developmentally appropriate which is fundamental to keeping this age group engaged.<br>It's just totally different. We tried gymnastics, swimming and they are all like I don't know how to explain it, just totally different. Like swimming's really structured and it gets a bit boring, whereas like the Rock Tots they do a warmup and then do some climbing and some games . . . It is so easy going. She doesn't get upset like when she has to hand her ribbon back at ballet. She just has to accept that she doesn't have to be quiet. It's just so easy going. (P2, Mother) **(6)**

			Provision of age-specific professionally supervised activities as a facilitator	when the kids are older it's easier you can drop them off for soccer and pick them up, but the little ones, you need to stay with them. **(11)**
			Age-group targeted opportunities as facilitator of participation	*we thought that Rock Tots being geared for her age group would be ideal for starting and getting her involved in it* **(6)**
**Community/Organisational**	**Affordability**	**Provision of low cost/free 'community' activities as a facilitator**	Low cost and novelty of activities as a facilitator of PA	*it was only recently that we started going bushwalking. And that just happened because a friend of mine called and wanted to take my daughter with her daughter to go bushwalking and I sort of went, "Hey, that's something we've not done together" and it was so cheap and easy. (Naomi)* **(7)**
			Lower Cost of PA programmes would facilitate participation for young families	introducing included lower cost programming for young families
			Limited availability of low-cost or free organised sport a barrier to Childs PA for fathers	fathers spoke about the limited availability of low-cost or free PA programs and organized sports for young children where they lived. **(10)**
			Lack of support from community councils' limits opportunities for community-based family PA	parents highlighted the lack of support from some community councils to make positive changes within the community negatively impacted their opportunity to co-participate in physical activity with their family. **(7)**
			Improved access to use facilities/resources already available in community as a potential facilitator of PA	the use of school facilities for younger families during non-school times:. . . *schools sit empty for a month-and-a-half at the end of the year, and I think that if they opened the doors for people to be doing activities there . . . it's just making use of the good resources that they already have available to them.* **(7)**
			Provision of free facilities would facilitate greater engagement in PA in deprived areas	there is the need for more free facilities for children' **(15)**

			Provision of free activities that incentivise the whole family important for child PA in deprived areas	Also, the involvement of 'family incentive exercise is imperative' (Se2, P1) and ideally there should be 'more active ... and free activities' (Se2, P1) 'for all of the family' (Se4, S1). A mixture of free swimming and team activities for all the family were mentioned as ways of improving participation in PA, to prevent obesity for preschool children and their parents. **(15)**
	Affordability	**Cost of attending formal provision as a barrier**	Cost as barrier to PA in deprived areas	the cost of family exercise was raised and it was stated that more activities need to be free Cost was also identified as a barrier, as parents perceived that they needed more money to facilitate PA. 'When the weather is not good, it is costly for swimming, play pits and structured activities' (Se3, P1). (se1, p2) **(15)**
			Cost of organised activities a barrier to Childs PA for fathers	*Our family earnings does not allow for enrolling her in swimming classes or gymnastics. We [parents] would like to have her enrolled in some type of activity but at the moment is not something we can afford* **(10)**
			High cost of organised sport a barrier to enrolment for fathers	Across all interviews In addition, several fathers reported that the high cost of organized sports and classes (e.g., swimming, ballet) was the main barrier that influenced their decision not to enrol their preschool-age children in these programs (e.g., soccer, swimming, ballet). **(10)**

			Cost of classes as a barrier to mothers enrolling children in classes	Furthermore, some mothers mentioned that limited resources kept them from enrolling their children in organized sports activities. One mother said: I'd like to enroll the little ones [children] in some classes, but sometimes it's expensive and the classes time are not good for my schedule . . . when the kids are older it's easier you can drop them off for soccer and pick them up, but the little ones, you need to stay with them. . . FGD #3; Mother #16 **(11)**
			Cost of activities and transport as a barrier to Childs PA	Safe options were often costly; indoor recreation centers (e.g., skating rinks) were expensive, as was the price of transportation **(12)**
			Cost prevents family participation and therefore Childs PA opportunities	obstacles such as high participation fees prevented family participation. **(13)**
			Parental financial constraints as a barrier to children PA	Some mothers spoke about not being able to provide child with opportunities for PA and active play due to financial constraints **(11)**
	Affordability	**Disposable income**	Lack of disposable income as a barrier to pre-schoolers PA	If parents had more disposable income, or if activities were cheaper/free, then parents stated they would promote PA and FMS development more with their preschool children **(15)**
			Fathers limited income as a barrier to Childs participation in sports, organised play and PA	In addition, fathers reported their limited finances as restricting their children's access to organized sports, organized play- time, and PA programs. **(10)**

			Limited income as a barrier to enrolment in organised (PA) activities for fathers	*With one income and having to pay for all the expenses there isn't much left for extras …* **(10)**
			Being able to afford to pay for child to attend facilitated attending 1:1 swimming lessons (middle-class parents)	*'We paid for one-to-one lessons for my son initially and in terms of cost obviously there is an additional expense there but when we've seen what she gets out of it I don't worry about cost. I certainly don't think Chloe [younger daughter] would have settled in a class like she has if we hadn't put her that in one-to-one first.'* One-to-one swimming lessons also placed their child at the centre of attention and ensured their child got specific attention, and these parents have the capital to put their child in a position of one- to-one learning. Parent 2 explains they do not worry about the cost because they have the economic capital to pay **(1)**<br><br>Middle-class parents feel they can absorb the extra cost in order to ensure their child is at the centre of the class and the classes are professional, formal and organised **(1)**
**Removed Codes**				
			The freedom of informal setting a facilitator for attending playgroup (? For mothers)	Overwhelmingly, parents and carers attend playgroup for social interaction and to receive support from other parents, so there was some concern that any formal program would negatively impact this. "This is probably one of the few places where I can come, and I can just leave him, because there's nowhere he can go, there's little he can destroy, and I can just either sit on the steps by myself and stare at nothing, or talk to other mums." Parent, FG5. **(4)**

