# Supplementary material for: Mixed-Methods Systematic Review to Identify Facilitators and Barriers for Parents/Carers to Engage Pre-School Children in Community-Based Opportunities to Be Physically Active
Source: Children (Basel). 2022 Nov 10;9(11):1727. doi: 10.3390/children9111727 (PMC9688361; doi:10.3390/children9111727)
Supplement: Supplementary file 1 [file children-09-01727-s001.zip › Table S1and S2.pdf]

children

MDPI

**Electronic Supplementary Material Breakdown of the quality assessments and supporting justification**

**Table S1.** Coding criteria for the Mixed Methods Assessment Tool.

| **Code Breakdown** | |
|---|---|
| Screening questions (For all types, if no, assessment should not continue) | S1. Are there clear research questions? |
| | S2. Do the collected data allow to address the research questions? |
| 1. Qualitative | 1.1. Is the qualitative approach appropriate to answer the research question? |
| | 1.2. Are the qualitative data collection methods adequate to address the research question? |
| | 1.3. Are the findings adequately derived from the data? |
| | 1.4. Is the interpretation of results sufficiently substantiated by data? |
| | 1.5. Is there coherence between qualitative data sources, collection, analysis and interpretation? |
| 2.Quantitative randomized controlled trials | 2.1. Is randomization appropriately performed? |
| | 2.2. Are the groups comparable at baseline? |
| | 2.3. Are there complete outcome data? |
| | 2.4. Are outcome assessors blinded to the intervention provided? |
| | 2.5 Did the participants adhere to the assigned intervention? |
| 3. Quantitative non-randomized | 3.1. Are the participants representative of the target population? |
| | 3.2. Are measurements appropriate regarding both the outcome and intervention (or exposure)? |
| | 3.3. Are there complete outcome data? |
| | 3.4. Are the confounders accounted for in the design and analysis? |
| | 3.5. During the study period, is the intervention administered (or exposure occurred) as intended? |
| 4. Quantitative descriptive | 4.1. Is the sampling strategy relevant to address the research question? |
| | 4.2. Is the sample representative of the target population? |
| | 4.3. Are the measurements appropriate? |
| | 4.4. Is the risk of nonresponse bias low? |

| | |
|---|---|
| | 4.5. Is the statistical analysis appropriate to answer the research question? |
| 5. Mixed methods | 5.1. Is there an adequate rationale for using a mixed methods design to address the research question? |
| | 5.2. Are the different components of the study effectively integrated to answer the research question? |
| | 5.3. Are the outputs of the integration of qualitative and quantitative components adequately interpreted? |
| | 5.4. Are divergences and inconsistencies between quantitative and qualitative results adequately addressed? |
| | 5.5. Do the different components of the study adhere to the quality criteria of each tradition of the methods involved? |

**Table S2.** Quality assessment results from Mixed Methods Assessment Tool.

| | **1. Qualitative Studies** | | | | | **2. Randomized Controlled Trials** | | | | | **3. Non-Randomized Studies** | | | | | **4. Quantitative Descriptive Studies** | | | | | **5. Mixed Methods Studies** | | | | | | |
|---|---|---|---|---|---|---|---|---|---|---|---|---|---|---|---|---|---|---|---|---|---|---|---|---|---|---|---|
| | 1.1. | 1.2. | 1.3. | 1.4. | 1.5. | 2.1. | 2.2. | 2.3. | 2.4. | 2.5. | 3.1. | 3.2. | 3.3. | 3.4. | 3.5. | 4.1. | 4.2. | 4.3. | 4.4. | 4.5. | 5.1. | 5.2. | 5.3. | 5.4. | 5.5. | **Overall Score** | **Comments** |
| Allen et al. (2021) | Y | Y | Y | Y | U | | | | | | | | | | | | | | | | | | | | | 4 | Difficult to tell for 1.4 and 1.5 because the results and discussion are combined, therefore data appears to be minimal, but there are quotes to support findings |
| Andrews et al. (2019) | Y | Y | U | U | U | | | | | | | | | | | Y | N | Y | N | Y | N | Y | Y | Y | N | 2 | 1.3, 1.4, 1.5 no details of thematic analysis approach, data collection, analysis or how themes derived. Quantitative & Qualitative data integrated. 4.3 unvalidated questionnaire but trialled prior to use. 4.4 risk of |

| | | | | | | | | | | | | | | | | | | | | | | | | | | | |
|---|---|---|---|---|---|---|---|---|---|---|---|---|---|---|---|---|---|---|---|---|---|---|---|---|---|---|---|
| | | | | | | | | | | | | | | | | | | | | | | | | | | | non-response bias from outer suburbs 5.1 no rationale for mixed method approach. 5.5 no information on quality /trustworthiness processes 5.4 no apparent divergences |
| Downing et al. (2016) | Y | Y | U | Y | Y | | | | | | | | | | | | | | | | | | | | | 4 | 1.3 lack of details on processes of thematic analysis, and trustworthiness of data analysis. Only qualitative data relevant. |
| Fuller et al. (2019) | Y | Y | Y | Y | Y | | | | | | | | | | | | | | | | | | | | | 5 | No identifiable issues |
| Grindley (2022) | Y | Y | Y | Y | Y | | | | | | | | | | | | | | | | | | | | | 5 | No identifiable issues |
| Grzywacz et al. (2016) | Y | Y | Y | Y | Y | | | | | | | | | | | | | | | | | | | | | 5 | No identifiable issues |
| Hnatiuk et al. (2020) | Y | Y | Y | Y | Y | | | | | | | | | | | | | | | | | | | | | 5 | No identifiable issues |
| Houghton et al. (2015) | Y | Y | Y | Y | Y | | | | | | | | | | | | | | | | | | | | | 5 | No identifiable issues |
| Joseph et al. (2019) | Y | Y | Y | Y | Y | | | | | | | | | | | | | | | | | | | | | 5 | No identifiable issues |

| | | | | | | | | | | | | | | | | | | | | | | | | | | | |
|---|---|---|---|---|---|---|---|---|---|---|---|---|---|---|---|---|---|---|---|---|---|---|---|---|---|---|---|
| Lindsey et al. (2022) | Y | Y | Y | Y | Y | | | | | | | | | | | | | | | | | | | | | 5 | No identifiable issues |
| Lindsey et al. (2019) | Y | Y | Y | Y | Y | | | | | | | | | | | | | | | | | | | | | 5 | No identifiable issues |
| Martin-Biggers et al. (2015) | Y | Y | Y | Y | Y | | | | | | | | | | | | | | | | | | | | | 5 | No identifiable issues. Only qualitative data relevant. |
| Penilla et al. (2017) | Y | Y | Y | Y | Y | | | | | | | | | | | | | | | | | | | | | 5 | No identifiable issues |
| Pisot (2020) | Y | Y | U | U | U | | | | | | | | | | | | | | | | | | | | | 2 | 1.3, 1.4, 1.5 No details on qualitative data analysis or interpretation. mixed method but only qualitative data relevant |
| Roscoe et al. (2017) | Y | Y | Y | Y | Y | | | | | | | | | | | | | | | | | | | | | 5 | No identifiable issues |
| Stirrup et al. (2015) | Y | Y | Y | U | U | | | | | | | | | | | | | | | | | | | | | 3 | Difficult to assess as the methodology definition is unclear. Authors themselves state that data is sparse |

Y = yes; N = no; U = unclear.
